# On the Influence of Opium as a Means of Preventing and Removing Some of the Injurious Consequences of Over-Work and Anxiety

**Published:** 1854-10

**Authors:** 


### ECLECTIC DEPARTMENT,

### AND SPIRIT OF THE MEDICAL PERIODICAL PRESS.

*On the Influence of Opium as a means of Preventing and Removing some of the Injurious Consequences of Over-work and Anxiety.* By Dr. Johnson, Assistant-Physician to King's College Hospital.

The following interesting and practical remarks are from a course of lectures on materia medica and therapeutics, delivered before the College of Physicians in 1853. Dr. Johnson proceeds:—

When all that is possible has been done for avoiding the cause of mental worry, and when all needful advice and encouragement has been given, we have next to direct our attention to the consequences, some of which will often continue long after their exciting cause has ceased to operate; while others are perpetuated by some persistent and unavoidable source of anxiety. Now, the first and the most frequent consequence of over-work or anxiety—the one too, which, more than any other, is productive of further mischief—is restlessness, or some form of disturbed and unrefreshing sleep. And the chief cure for this, after the causes have been as much as possible avoided, is an opiate at bedtime. So far as I can see, it is of little importance what preparation of opium or of morphia is used. For hospital patients I generally order the compound soap-pill; one advantage of which is, that its name does not indicate its opiate nature. The dose must vary according to circumstances. In ordinary cases five grains, of the pill, that is, one grain of opium, may be taken every night at bedtime. In a case of much excitement, with extreme restlessness or a threatening of delirium, the dose must be double or treble that which I have mentioned. In such cases, however, the opium would be best given in a liquid state—in the form of tincture, or the solution of the muriate or acetate of morphia.

The time for the continued exhibition of the opiate must vary according to circumstances, and will be much influenced by the success of the treatment. The object is to break the habit of dreaming restlessness, and to procure sound and refreshing sleep. In many cases this object may be attained by the nightly repetition of the dose for one week. It is seldom necessary or desirable to continue the medicine for more than a month, though in some cases, it may be expedient and beneficial to extend the period considerably. In many cases I have found that the beneficial effects of the medicine have been immediate; the patient has slept soundly, the distressing dreams have ceased, the appetite has returned, and all the symptoms which depended on loss of sleep and loss of appetite have quickly disappeared. After a few nights of sound sleep have been procured by the opiate, the dose should be discontinued, and in most cases the patient will continue to sleep as well without the medicine as with it. There is, probably, no one medicine which has the power of quickly removing such a multitude and a variety of distressing symptoms as opium, when its action is really favorable in the cases to which I refer. It is not, however, to any specific efficacy residing in the opium, but to the marvelous influence of sleep in refreshing both body and mind, that the benefit is really due. The value of the opiate consists in the fact, that, on the whole, it is the safest and most certain means of procuring sound sleep.

The use of opium as a medicine is sometimes attended with unpleasant consequences, and it does not always effect what is desired. I proceed now to indicate some of the unfavorable results of the opiate treatment, and the precautions which ought to be observed in the use of the medicine. One of the most frequent discomforts attending the use of opium is a feeling of nausea and faintness, either with or without headache, in the morning after waking. The best cure for this is a cup of coffee or tea, with some solid food, followed by a walk in the open air. In many cases the opium, although at first it may disagree, yet produces no unpleasant effect after the second or third dose.

The nervous patients who require the method of treatment which I am advocating, almost invariably suffer from constipation,—a torpid condition of the bowels, being, in fact, one of the natural consequences of the general debility which characterizes the patients in question. Although the immediate effect of the opium is to increase the constipation, yet its ultimate tendency is to restore the regular action of the bowels, by means of the invigorating influence derivable from sound refreshing sleep, and an increased appetite for food. The temporary constipation may readily be obviated by an occasional mild aperient—a seidlitz powder, or a compound rhubarb or colocynth pill. The inconvenience arising from the astringent effect of opium upon the bowels is so easily met and removed, that it would never deter me from giving the medicine in any case which appeared to require it.

One of the most serious objections to the use of opium, is its tendency, in some cases, to produce an effect the direct opposite of that which we require,—to produce wakefulness and excitement, instead of sleep and composure. It is only in a small proportion of cases that this difficulty arises. It may sometimes be overcome by changing the form of the medicine, or by increasing the dose of the opium or morphia, and, in other cases, by combining the opiate with a moderate dose of antimony—James's powder, or tartar emetic—a combination which has been strongly recommended by Dr. Graves to procure sleep and check delirium in some cases of fever. It must, however, be admitted, that some patients cannot tolerate opium in any form or in any dose; and nothing can better show the value of this drug than the difficulty of finding a substitute for it. We may try henbane and hop, and these will sometimes effect our object; but their action is very uncertain in comparison with that of opium.*

It is well to remember that an opiate enema will sometimes procure refreshing sleep, when opium, in any form administered by the mouth, is either quite inoperative, or productive only of distressing excitement or sickness.

But may not the frequent repetition of an opiate dose become a necessity for the patient? May we not be instrumental in making him an opium-eater? I admit that the danger of such an evil, if real, would be a very fearful one. There are few results of medical practice which I should regret more than the reflection, that I had in any way contributed to render a recourse to narcotics or stimulants habitual or necessary to a single patient. I believe, however, that a cautious use of opium is attended with little danger of leading to so terrible an abuse of the drug.

In giving opium to hospital patients, I never tell them what they are taking; and one reason for preferring the compound soap-pill, in such cases, is, as I have before intimated, that the nature of the medicine is not apparent from the prescription, if the patient should read it. The opium should be discontinued as soon as it can be dispensed with,—as soon, that is, as restlessness and frightful dreams have ceased to harass and exhaust the patient. The rapid convalescence, and the renewed health, and strength, and spirits, which are wonderfully promoted by securing sound and refreshing sleep, will generally enable the patient at once, and without difficulty, to dispense with the use of opiates. I should withhold opium from a patient who neglects any directions which I have given him as to exercise, diet, and the general management of himself, and whose restlessness and nervousness appear to result from such negligence. In other words I would not encourage a patient to trust habitually to opium for the removal of discomforts which might be avoided by the exercise of self-control, and by obedience to natural laws.

I beg to make an earnest protest against the routine practice of giving opiates to every patient who complains of inability to sleep. Our first care

---

*Since this lecture was delivered, I have found reason to believe that one of the best substitutes for opium in the cases refered to, is chloroform, in doses of fron *m* x to *m* xx, made into a draught with mucilage.

must be to discover, and then to remove the cause of the sleeplessness. We shall meet with some indolent patients, for whom the best soporific is regular employment and daily active exercise in the open air; for others, who are feeble, tonics and nutritious food will be the appropriate remedies; and again, in other cases, dyspeptic symptoms will cease, and refreshing sleep will return, under the influence of an occasional aperient and carefully regulated diet. In most cases of this kind, the exhibition of opium would not only be unsuccessful, but positively hurtful.

The cases in which the opiate treatment is most rapidly and completely successful are those in which the nervous symptoms are the result of some past grief, or anxiety, or fatigue, the impression of which remains, and is perpetuated by the patient's inability to obtain refreshing sleep. In such instances, a few nights of sound sleep, procured by means of the opium, rarely fail to effect a rapid cure, and this, too, after the nervous symptoms have continued for many months, or even for years.

Another class of cases in which equal benefit is often derived from a similar method of treatment, are those in which nervous restlessness has been induced by continued overwork, whether mental or bodily. In such instances, it is obviously desirable, as I have before intimated, that the patient should rest, or diminish his labors, if possible; but the patient may assure us that he has no alternative but to go on with his work, or to lose his employment, and with it his means of living. In such a case, we may often prevent overworked men and women from breaking down, and enable them to go on in comparative comfort, by giving an opiate nightly for a week or two. Refreshing sleep will be induced, the appetite will return, and, as a consequence, the strength and spirits will revive. And the strength and spirits thus obtained are not false and artificial in the same pernicious way as the stimulus obtained from alcohol, by which too many are tempted in the circumstances to which I have referred. The temporary help which a languid body or mind derives from alcohol is generally followed by a corresponding amount of depression, and with this there comes a craving for a repetition of the stimulant. Another bad result of the too free use of alcohol is a loss of appetite and an impaired power of digestion. Now, the effects of the opiate plan of treatment, conducted with the precautions to which I have before alluded, are in most respects the opposite of those produced by alcoholic stimulants; for we seek, by means of opium, a natural remedy for fatigue, that remedy being sleep, which brings with it a desire for food, and the power to digest it. Alcohol is taken for the sake of the immediate stimulus; the subsequent depression is the drawback upon its utility as a means of keeping up the working powers. The object in giving opium is to obtain, not its stimulant effects, which are comparatively slight and transient, nor immediately its composing influence, but the refreshment which follows the latter, and which has nothing corresponding with it among the ordinary consequences of alcoholic stimulants.

My objections to the *abuse* of alcohol as a stimulant do not, of course, apply to the use of wholesome wine and beer as articles of diet by those who require them, and who appear to derive benefit from them. Moreover, there are certain cases of nervous disease in which some form of alcoholic stimulant may be given with great advantage, either alone or in conjunction with opium. I refer to cases of extreme restlessness, either with or without delirium, and whether resulting from intemperance or from grief, or watching or fatigue, when the bodily powers are very feeble, although under the mental influence there may be great excitement. In these cases repeated large doses of opium sometimes fail to procure sleep, but appear rather to have a depressing influence: the patient's skin becomes cold, and is bathed in perspiration, while the delirium and excitement continue. In such circumstances, the continued use of the opium is not only useless, but injurious and dangerous; and the surest mode of arresting the collapse, and of procuring sleep, is to give freely either wine or brandy, or in cases of intemperance, the stimulant to which the patient has been accustomed, with beef-tea, or some other form of nourishment.

It is scarcely necessary to observe, that in all cases of nervous disease we must carefully watch the signs of functional disturbance or of structural change in any organ of the body, and that we must meet such symptoms by the appropriate remedies. And although, in most instances, a tonic plan of treatment is required, yet we must not hesitate to resort to measures of depletion if they are called for by the occurrence of such organic disease as appears to need this treatment.

The cases which are least favorable either for the opiate or for any other plan of treatment are: 1st, cases of confirmed hypochondriasis or melancholy of very long duration, and especially when these have the character of religious despondency; 2dly, cases in which extreme nervousness has resulted from great terror, or from a sudden shock, which has left a deep and durable impression upon the mind and nervous system; and lastly, cases in which the symptoms are perpetuated by some constant source of anxiety or sorrow.

These classes of cases, although very unfavorable, and often little benefited by any plan of treatment, whether medical or moral, are yet by no means hopeless nor always incurable. Their unfavorable and unmanageable character is, however, greatly confirmed when they are complicated with epilepsy; and this whether the epilepsy has been induced by a sudden shock of grief or terror, or whether it has supervened upon long-continued anxiety and nervousness.

